# Combined Analysis of Methylation and Gene Expression Profiles in Separate Compartments of Small Bowel Mucosa Identified Celiac Disease Patients’ Signatures

**DOI:** 10.1038/s41598-019-46468-2

**Published:** 2019-07-10

**Authors:** D. Cielo, M. Galatola, N. Fernandez-Jimenez, L. De Leo, K. Garcia-Etxebarria, C. Loganes, A. Tommasini, T. Not, R. Auricchio, L. Greco, J. R. Bilbao

**Affiliations:** 1 Department of Translational Medical Sciences, University of Naples “Federico II”, Naples, Italy; 2 European Laboratory for the Investigation of Food Induced Diseases (ELFID), University of Naples “Federico II”, Naples, Italy; 3 Department of Genetics, Physical Anthropology and Animal Physiology, University of the Basque Country (UPV-EHU), BioCruces Health Research Institute, Leioa, Spain; 4 Institute for Maternal and Child Health, IRCCS “Burlo Garofolo”, Trieste, Italy

**Keywords:** Gene expression, DNA methylation

## Abstract

By GWAS studies on celiac disease, gene expression was studied at the level of the whole intestinal mucosa, composed by two different compartments: epithelium and *lamina propria*. Our aim is to analyse the gene-expression and DNA methylation of candidate genes in each of these compartments. Epithelium was separated from *lamina propria* in biopsies of CeD patients and CTRs using magnetic beads. Gene-expression was analysed by RT-PC; methylation analysis required bisulfite conversion and NGS. Reverse modulation of gene-expression and methylation in the same cellular compartment was observed for the IL21 and SH2B3 genes in CeD patients relative to CTRs. Bioinformatics analysis highlighted the regulatory elements in the genomic region of SH2B3 that altered methylation levels. The cREL and TNFAIP3 genes showed methylation patterns that were significantly different between CeD patients and CTRs. In CeD, the genes linked to inflammatory processes are up-regulated, whereas the genes involved in the cell adhesion/integrity of the intestinal barrier are down-regulated. These findings suggest a correlation between gene-expression and methylation profile for the IL21 and SH2B3 genes. We identified a “gene-expression phenotype” of CeD and showed that the abnormal response to dietary antigens in CeD might be related not to abnormalities of gene structure but to the regulation of molecular pathways.

## Introduction

Celiac disease (CeD) is a systemic immune-mediated disease triggered by gluten ingestion in genetically susceptible individuals. It is the most common form of food intolerance, and its prevalence has increased over the last three decades^1^.

CeD has a strong genetic component, as suggested by our twin studies^2^. The primary genes associated with CeD are Major Histocompatibility Complex class II (MHC-II) genes encoding HLA-DQ2 (i.e. HLA-DQA1*05 and HLA-DQB1*02) or HLA-DQ8 (i.e. HLA-DQA1*03 and HLA-DQB1*03:02). These molecules consists of an alpha chain (HLA-DQα) and a beta chain (HLA-DQβ) that form a heterodimer, which is anchored to the cell membrane. There is an HLA gene-dose effect on disease risk, as individuals carrying two copies of HLA-DQ2 have a higher susceptibility for celiac disease than do those with only one copy^3^. Recently, it was shown that HLA-DQ7 represents an additive or independent CD-risk haplotype with respect to HLA-DQ2/DQ8 haplotypes^4^. However, these haplotypes are common in the general population, and not all carriers develop clinical disease; thus, they are not sufficient for disease development, accounting for approximately 40% of the heritability of CeD^5^.

Both MHC and non-MHC genetic factors influence CeD development, and since the first case/control Genome-Wide Association Study (GWAS) on celiac disease was published in 2007^6^, a total of 57 non-HLA loci have been identified as associated with this disease. To date, 57 non-MHC variants have been estimated to account for 15% of CeD heritability, but the remaining 50% heritability of CeD remains unexplained^7^.

In a previous study, we aimed to improve the estimation of CeD risk in siblings by adding to HLA haplotype a small set of non-HLA genes. Applying a Bayesian approach, we improved the estimation of CeD risk among siblings over the HLA-based risk, providing a tool to predict the disease in at-risk individuals^8^.

The vast majority of CeD-associated SNPs do not map to exons but intersect with regulatory regions, implying that protein changes do not govern disease development. The analysis of expression Quantitative Trait Locus (eQTL) CeD-associated polymorphisms has shown that these anomalies often affect the expression of nearby genes in different cell types^9,10^; however, to date, they have been explored in small intestinal biopsiesonly, which contain multiple cell types, leading to results that are difficult to interpret. The intestinal mucosa is composed of two different compartments, the epithelium and the *lamina propria*.

The immune responses in the epithelium and *lamina propria* are separated by a basement membrane, which appears thinner and with more breaches in patients with active CeD than in patients on gluten-free diets or in non-CeD subjects^11^. The upstream events activating adaptive immune responses that occur in the *lamina propria* interact with the downstream events in the epithelium. However, how these immune responses in the *lamina propria* and the epithelium interact remains unclear^12^.

In our previous work, we showed that the combined expression of 4 non-HLA selected genes in peripheral blood monocytes enabled discrimination between CeD patients and controls (CTRs) and between CeD patients on a gluten-free diet and disease controls^13^. We then confirmed the importance of gene expression by showing that the expression of a small set of candidate genes, in peripheral blood mononuclear cells, can predict CeD at least 9 months before the appearance of any clinical and serological sign of disease in genetically at-risk infants^14^.

In the present study, we aim to analyse a set of candidate genes to explore both genetic and epigenetic alterations in isolated intestinal cell populations from both the epithelium and *lamina propria*.

The final aim of this work is to evaluate alterations in candidate gene expression; identify, at the level of distinct cell populations, potential alterations consistent with the gluten-induced damage in CeD; and describe the mechanisms of epigenetic regulation that underlie these alterations.The final aim of this work is to evaluate alterations in candidate gene expression; identify, at the level of single and distinct cell populations, potential alterations consistent with the gluten-induced damage in CeD; and describe the mechanisms of epigenetic regulation that underlie these alterations.

## Results

### Evaluation of sample purity

The efficacy of separation between the two compartments, epithelium and *lamina propria*, was evaluated by real-time PCR. We measured the Epithelial Cell Adhesion Molecule (EpCAM) expression level, as the specific marker of the epithelial cells, in both epithelial and *lamina propria* cells, normalized to the expression of an endogenous gene (GUSb) and used as reference sample in the epithelial compartment. As shown in Supplementary Fig. 1, a 98% purity of the epithelial compartment was achieved. In particular, the analysis of EpCAM expression generated a selection of 97.8% epithelial cells (CD326+) in celiac biopsies and 97.5% epithelial cells (CD326+) in the biopsies of controls (Supplementary Fig. 1).

### Gene expression in the epithelium and *lamina propria*

The expression of 16 CeD-associated genes (Table 1) in each compartment was compared between CeD patients and controls. To simplify interpretation, we grouped the candidate genes into 4 putative functional groups: (1) genes directly involved in inflammation and damage, (2) “classical” candidate genes strongly associated with CeD of unpredictable function, (3) genes involved in the regulation of inflammation and damage, and (4) genes involved in the maintenance of cell adhesion and intestinal barrier integrity. These genes were selected based on their robust replication in several GWASs, our previous studies in several models and their likely putative roles in the gluten-induced abnormal immune response^6–10,13,14^.

**Table 1:** List of genes analysed in the study, their functions, and the TaqMan Gene Expression assays used in the expression experiments (Life Technologies).

Gene	Function	Functional Group	TaqMan Assay
IL12A	Pro-inflammatory cytokine	Inflammation/Damage Direct	Hs00222327_ml
IL21	Pleiotropic cytokine	Inflammation/Damage Direct	Hs00168405_ml
NFKB1	Regulation of autoimmunity and inflammation	Inflammation/Damage Direct	Hs00765730_m1
C-REL	Subunit of the NF-kB transcription complex	Inflammation/Damage Direct	Hs00968436_m1
TNFAIP3	Negative feedback loop control of NF-kB	Inflammation/Damage Direct	Hs00234713_m1
KIAA1109	Located in the genomic region associated with CeD	Canidated/Associated	Hs00361070_ml
SH2B3	Activates PI3-kinase	Inflammation/Damage Regulation	Hs00193878_m1
RGS1	Lymphocytes Homing	Inflammation/Damage Regulation	Hs00175260_m1
TAGAP	Negative regulator of the immune response	Inflammation/Damage Regulation	Hs00611823_m1
TNFRSF14	Activation of NK intestinal and CD4 + T cells, “gut-homing cells”	Inflammation/Damage Regulation	Hs00998604_ml
TNFSF14	Activation of NK intestinal and CD4 + T cells, “gut-homing cells”	Inflammation/Damage Regulation	Hs00542477_m1
LPP	Extracellular matrix and cell-cell contact homeostasis	Cell Adhesion/Integrity of Intestinal Barrier	Hs00944352_m1
TJP1	Proteins of the tight junctions involved in maintaining the integrity of the intestinal barrier	Cell Adhesion/Integrity of Intestinal Barrier	Hs01551861_ml
PTPRK	Maintenance of cell junctions and participation in the modulation of EGFR activity, resulting in an inhibition of cell proliferation	Cell Adhesion/Integrity of Intestinal Barrier	Hs00267788_ml
ARHGAP31	Regulation of cell migration, focal adhesion size and dynamics	Cell Adhesion/Integrity of Intestinal Barrier	Hs00393361_ml
C1orf106	Involved in cell adhesion processes	Cell Adhesion/Integrity of Intestinal Barrier	Hs01009089_ml

**Figure 1:**
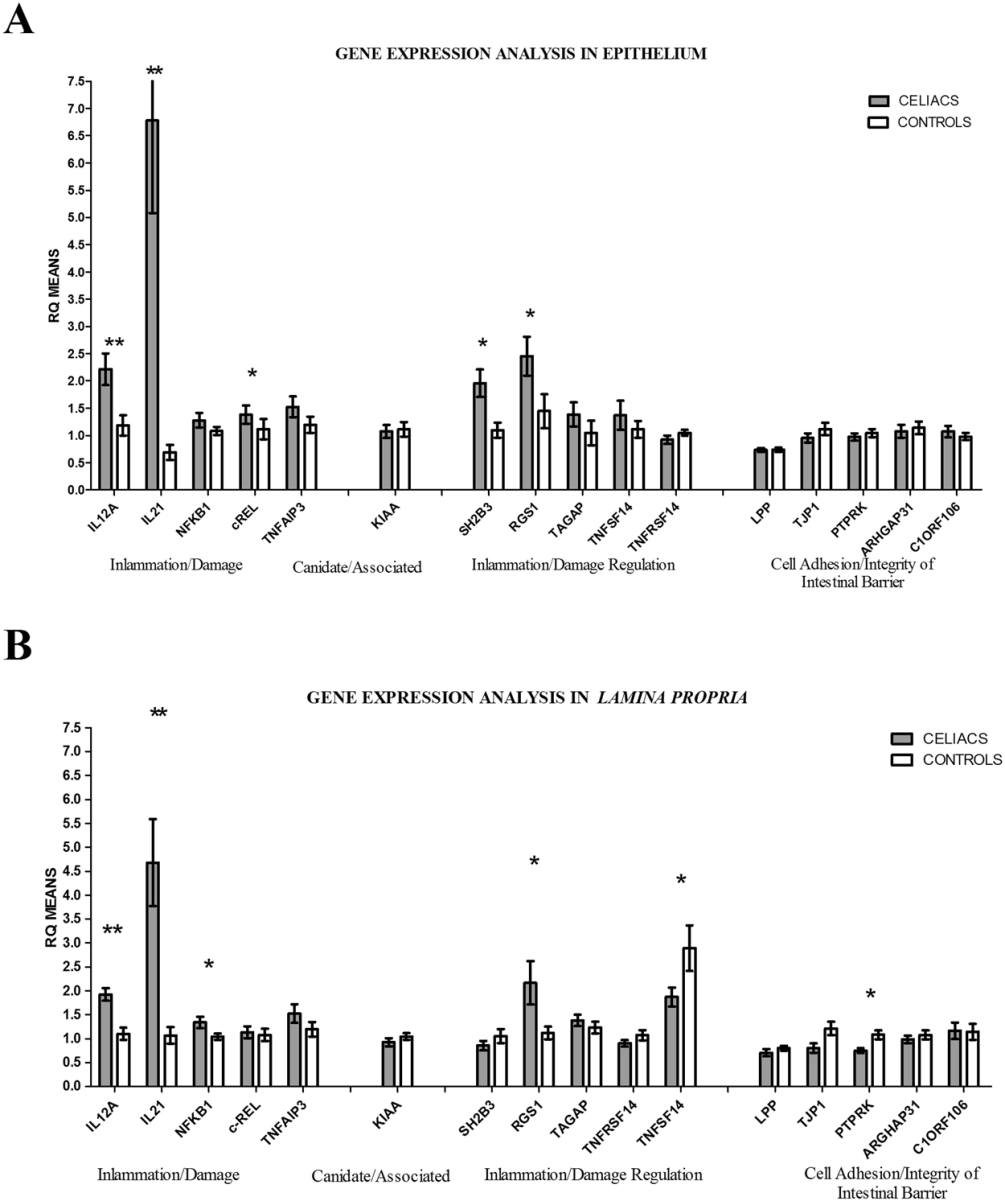
Gene expression analysis. In each of the epithelium and *lamina propria*, expression of 16 CeD-associated genes was compared between CeD patients and CTRs. The IL12A, IL21, c-REL, RGS1, and SH2B3 genes were significantly up-regulated in the epithelial cells of CeD patients relative to CTRs. In the *lamina propria*, the IL12A, IL21 and RGS1 genes were equally upregulated between CeD patients and CTRs. In addition,TNFSF14 and PTPRK were down-regulated in CeD patients relative to CTRs (**B**). *p < 0.05; **p < 0.01.

Figure 1A shows that the *IL12A*, *IL21*, *c-REL*, *RGS1*, *SH2B3* genes, which are directly or indirectly involved in the inflammation process, were significantly up-regulated in the epithelial cells of CeD patients relative to controls.

Similarly, IL12A, IL21, NFKB1 and RGS1 were equally up-regulated in the *lamina propria* of CeD patients relative to their corresponding expression in controls. In addition,TNFSF14 and PTPRK were down-regulated in the *lamina propria* of CeD patients relative to their expression in controls (Fig. 1B). Figures of the expression of each gene are provided in the supplementary data (Supplementary Fig. 2).

Differences between CeD patients and controls were evaluated by the rank-sum test (Mann-Whitney) because of the asymmetry in the data distributions.

### Correlations in gene expression

The expression of genes in each compartment is the result of complex correlations among genes within specific and inter-related metabolic and signalling pathways.

Thus, it was of interest to examine the correlations among genes within a specific compartment in CeD patients and controls.

**Figure 2:**
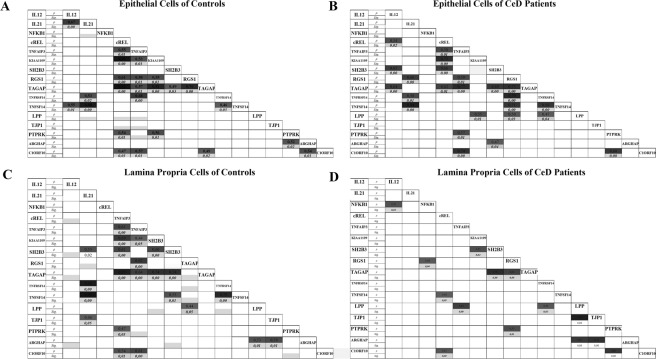
Patterns of correlations between genes in expression in the epithelium (**A**,**B**) and *lamina propria* (**C**,**D**) of celiacs and controls. For each pair of genes, the intensity of coloration of the box is proportional to the correlation between them, and the Pearson coefficient (ρ) is shown. Only those correlations significant at *p* < 0.05 are shown.

The results demonstrate the complexity of these correlations, which include not only bivariate correlations but also multi-dimensional correlations. Figure 2 shows the correlations of gene expression in the epithelium of CeD patients (B) and controls (A) and in the *lamina propria* of controls (C) and CeD patients(D).

It may be observed that, based on the degree of darkness, some metabolic profiles were well correlated in both CeD patients and controls in both compartments, reflecting “mandatory” functional pathways. In contrast, other correlations that were strong among controls were absent in the epithelium and *lamina propria* of CeD patients. In the *lamina propria* compartment, 24 strong correlations (correlation coefficient (r) values > 0.5) were observed in the controls, whereas only 14 such correlations were observed in CeD patients. Specifically, in the *lamina propria* of controls, the expression of TNFAIP3 was strongly correlated with the expression other genes involved in the regulation of inflammation, including TAGAP, RGS1, cREL and C1orf106. In the *lamina propria* of CeD patients, there were no significant correlations among these genes, although a correlation between TNFAIP3 and LPP expression was observed.

In contrast, in the epithelium of CeD patients, no correlations were lost relative to those observed in the epithelium of controls, but several candidate genes showed stronger correlations in CeD patients than in controls. SH2B3 was strongly correlated with TAGAP, cREL and IL-12A only in CeD patients, suggesting a specific role of this gene in the gluten-induced immune response.

### Signatures of CeD in the epithelium and *lamina propria* as evidenced from multivariate analysis

Multivariate analysis was performed to better understand the differential involvement of the candidate genes in the two compartments between CeD patients and CTR subjects.

Tables 2 and 3 show the results of the discriminant analysis for the epithelium: a small set of genes (IL21, TNFSF14 with TNFRSF14, NFKB1 and SH2B3) discriminated most CeD patients (13/18; 72.2%) from controls, whereas only 2/18 controls (11.1%) were incorrectly classified as CeD patients.

**Table 2:** Stepwise discriminant analysis of gene expression in epithelial cell in epithelial cells.

Step	Gene	Wilk’s Lambda	Variance Ratio F	
			Statistic	*p*
1	IL21 EPI	0,749	11,042	0.000
2	TNFSF14 EPI	0,643	8,878	0.000
3	NFKB1 EPI	0,558	8,183	0.000
4	TNFRSF14 EPI	0,508	7,268	0.000
5	SH2B3 EPI	0,456	6,926	0.000

Five genes (IL21, TNFSF14, NFKB1, TNFRSF14, and SH2B3) were selected for analysing discrimination capacity, with a *p* value less than 0.001.

**Table 3:** Classification by discriminant equation of gene expression in epithelial cells.

	Status	Predicted Group		Total
		CD	Not CD	
Original Group	**CeD**	13 (72.2%)	5(27.8%)	18
	**Not CeD**	2 (11.1%)	16 (88.9%)	18

Results of the prediction analysis: 88.9% of controls and 72.2% of celiac patients were correctly classified. Overall Correct Classification = 80.6%

In the *lamina propria* (Tables 4–5), the combinations of IL12 and IL21, TNFSF14 and PTPRK, and NFKB1 and KIAA1109correctlyclassified 90% (16/18 CeD patient and 17/19 CTRs)of individuals.

**Table 4:** Results of discriminant analysis in *lamina propria* cells.

Step	Gene	Wilk’s Lambda	Variance Ratio F	
			Statistic	*p*
1	IL12 LP	0,595	23,100	0.000
2	NFKB1 LP	0,442	20,823	0.000
3	TNFSF14 LP	0,400	16,018	0.000
4	IL21 LP	0,314	16,949	0.000
5	PTPRK LP	0,288	14,814	0.000
6	KIAA1109 LP	0,270	13,040	0.000

Six genes (Il12, NFKB1, TNFSF14, IL21, PTPRK, and KIAA1109) were selected for analysing discrimination capacity, with a *p* value less than 0.001.

**Table 5:** Classification by discriminant equation of gene expression in *lamina propria* cells.

	Status	Predicted Group		Total
		CD	Not CD	
Original Group	**CeD**	16 (88.9%)	2 (11.1%)	18
	**Not CeD**	2(10.5%)	17 (89.5%)	19

Results of the prediction analysis: 89.5% of controls and 88,9.2% of celiac patients were correctly classified. Overall Correct Classification = 89.2%.

TNFSF14 and IL21 with NFKB1 were efficient discriminators in both compartments. In the epithelium, the receptor of TNFSF14 (TNFRSF14) and SH2B3 provided further discrimination. In the *lamina propria*, PTPRK, KIAA1109 and IL12 contributed to better discrimination.

However, the actual “best profile”, obtained through a mathematical procedure, should be interpreted under a functional scenario. The specific pattern of variable selection through the stepwise procedure in the epithelium is not shown (Supplementary Table 1). At step 0, the gene producing the best F (variance) ratio between the CeD patients and CTRs was selected (IL21 F = 11.04): the second-best gene was IL12 (F = 8.22). However, at step 1, having included IL21 in the model, the F Ratio of IL12 decreased to 2.4. Thus, IL12 lost its ability to contribute to the discriminant model but certainly did not lose its function. In the *lamina propria*, RGS1 was a strong discriminator at step 0 (F = 4.6), but after the best gene,IL12, was included (with F = 23.1), RGS1 completely lost its discriminant ability (F declined to 0.35). However, it certainly did not lose its functional significance (Supplementary Table 2).

**Figure 3:**
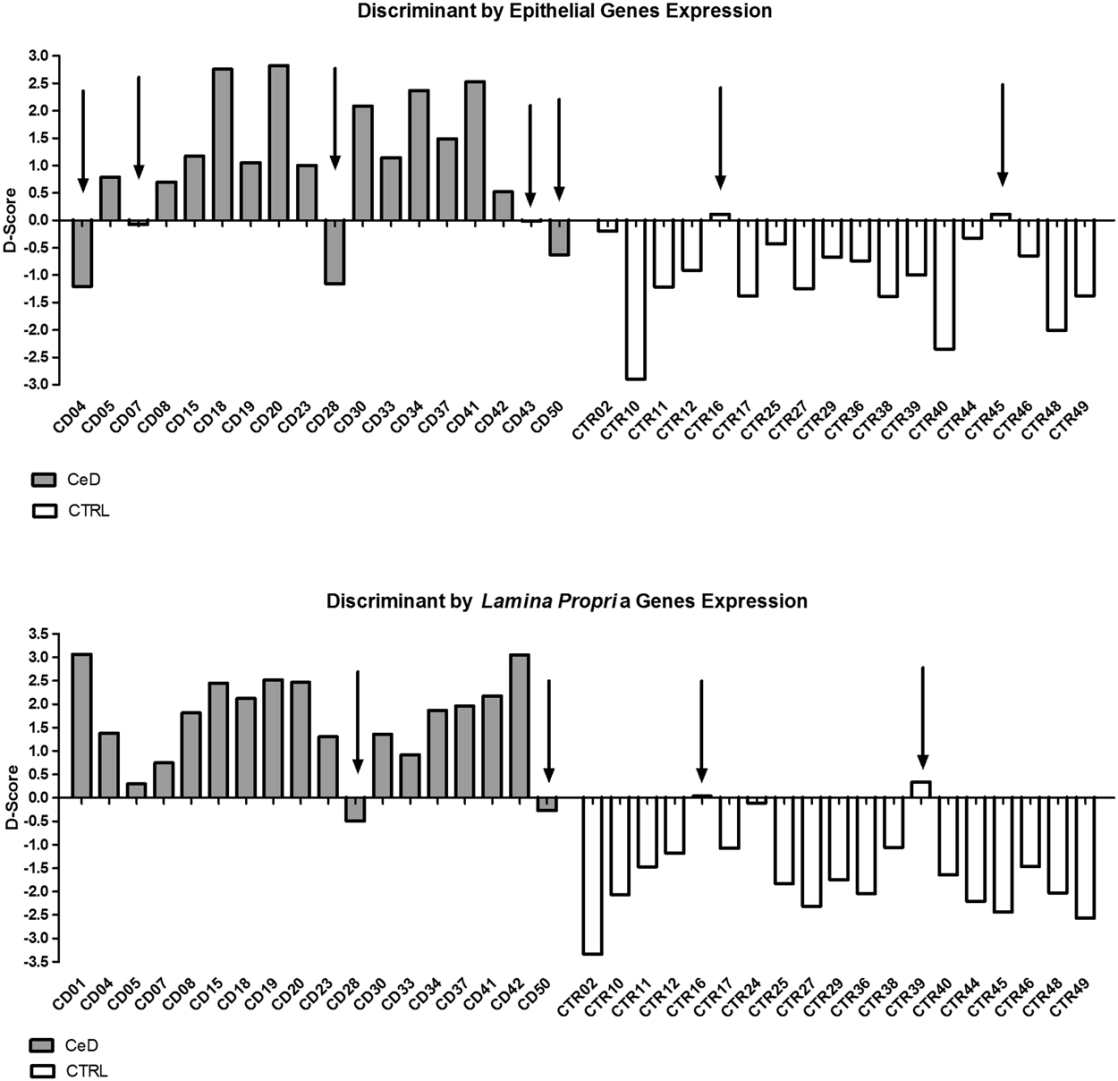
D-Score graphs. For the epithelial cells, five CeD and two non-CeD samples (indicated by the arrows) were misclassified, yielding a total correct classification rate of 80.6% (**A**). For *the lamina propria* cells, two CeD and two non-CeD samples were misclassified, yielding a total correct classification rate of 89.2% (**B**).

Through the model obtained by the analysis, a discriminating score (D-Score) was computed for each individual, related to the probability of membership among the cases or controls. Figure 3A,B show the pattern of probability of membership according to D-Score in CeD patients and controls. The gene expression signature obtained by the analysis produced an acceptable distinction of the celiac children from the controls. The classification might be slightly optimistic since we classified individuals by the coefficients obtained in the same cohort. However, when we applied a jack-knife method, classifying each individual through an auto-exclusion procedure, we still obtained 70% correct classification.

### Methylation analysis

**Figure 4:**
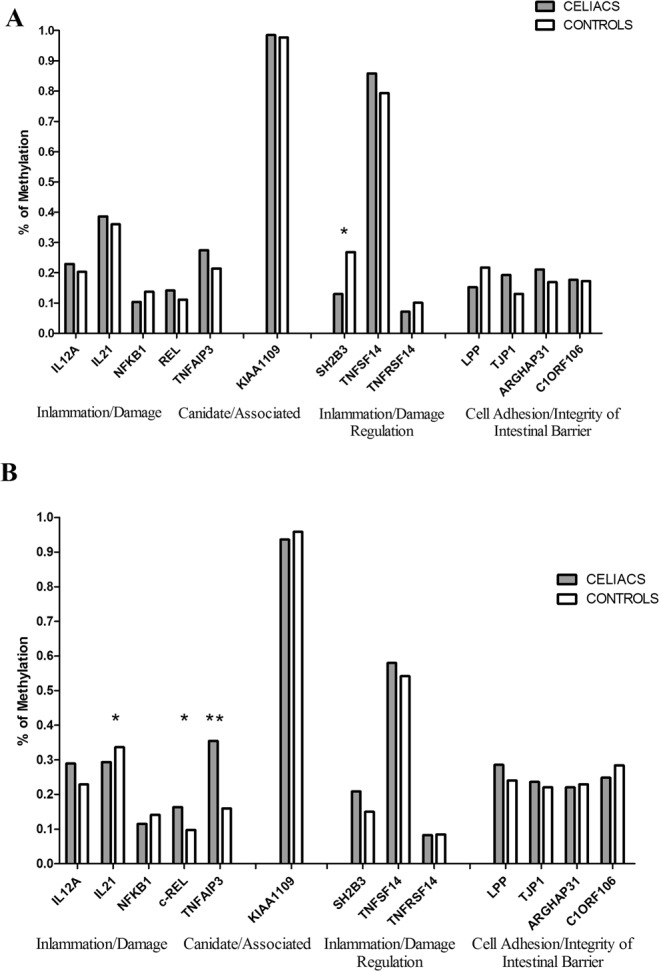
Average level of methylation for the candidate genes in the epithelium and *lamina propria*. In the epithelial cells (**A**), only SH2B3 was differentially methylated between CeD and CTR subjects (*p* = 0.003), whereas in the *lamina propria* (**B**) the genes IL21 (p = 0.03), TNFAIP3 (p < 0.001) and cREL (*p* = 0.005) showed differences in methylation level between CeD patients and CTRs. *p* < 0.05; **p < 0.01

The methylation analysis revealed several differences between CeD patients and controls in each compartment. Unfortunately, for RGS1 and PTPRK genes, the list of CpG islands was not available: they were not included in the methylation analysis. The differences in “mean level of methylation” for all candidate genes are shown in Fig. 4A,B. A mean value may show an aliasing bias since the methylation of specific CpG islands of the gene might be more important in regulating gene expression than is the average methylation through at least 20–40 CpG islands.

Figure 4A,B shows the average level of methylation of the candidate genes in either the epithelium or the *lamina propria*. In epithelial cells, only SH2B3 was differentially methylated between CeD patients and controls (*p* = 0.003), whereas in the *lamina propria*, the genes IL21 (*p* = 0.03), TNFAIP3 (*p* < 0.001) and cREL(*p* = 0.005) showed differences in methylation level between CeD and CTRs.

**Figure 5:**
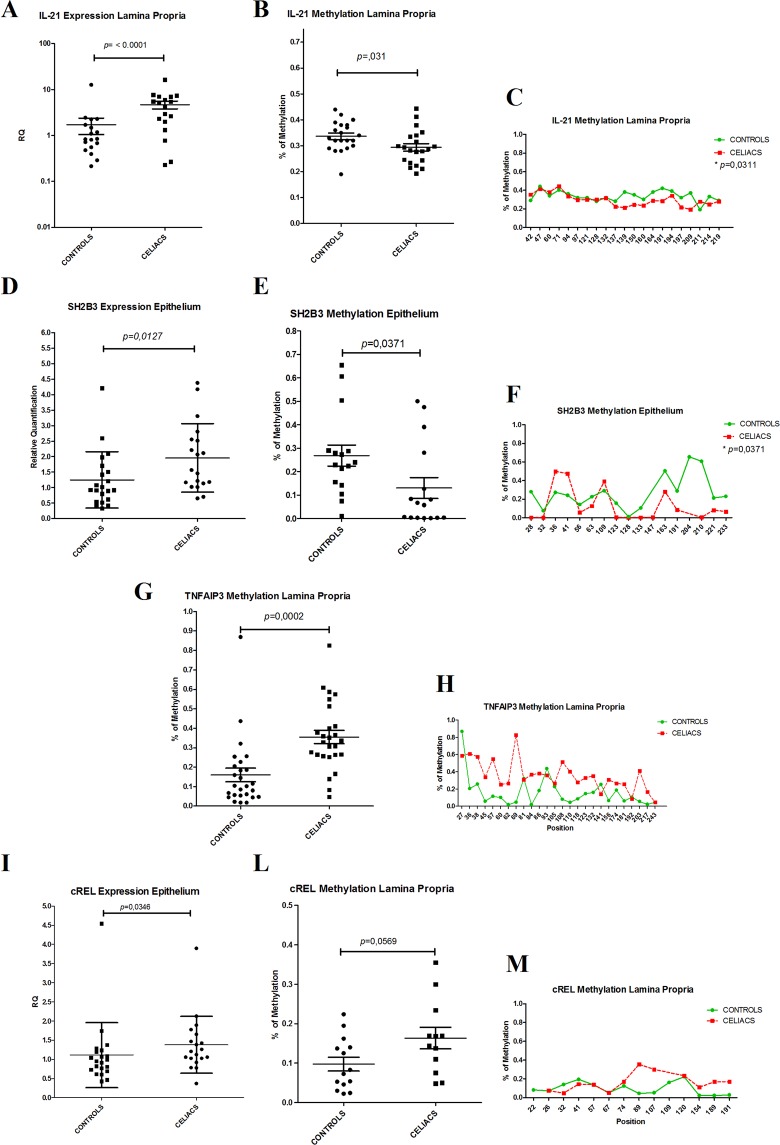
Fine representation of methylation levels across the genes sequences. IL21(panel A) and SH2B3 (panel D) showed enhancer gene expression associated with lower methylation of the genes (panels B and E) in CeD patients than in controls. Conversely, TNFAIP3 (panel G) and cREL (panel L) showed higher methylation levels in CeD patients than in controls; higher gene expression in CeD patients was observed only for cREL. The methylation of each single nucleotide of the CpG island (position) is shown in panels C,F, H, and M.

Further investigation showed that IL21 expression in the *lamina propria* was greater in CeD patients than in controls (Fig. 5A):this gene was 20% less methylated in CeD patients than in controls. Figure 5B shows that several regions of the gene were differentially methylated. Figure 5C shows the methylation of each single nucleotide of the CpG island.

**Figure 6:**
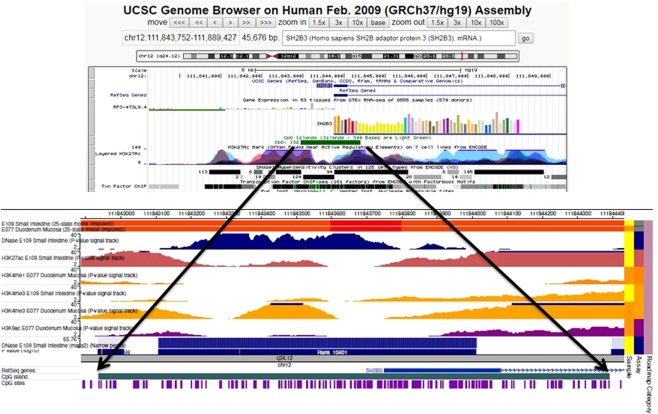
Bioinformatics analysis output related to the regulation elements of the SH2B3 gene: (**A**) Ref Seq analysis showed the presence of regulation elements in the SH2B3 gene region evidenced by the alteration of methylation levels in the duodenal mucosa. (**B**,**C**) Data analysed by the Epigenome Roadmap tool showed the presence of regulation elements DNase, H3K27ac, H3K4me3, and H3K9ac in the region.

Similarly, the lower methylation of the SH2B3 gene in CeD epithelial cells was associated with higher expression of the gene (Fig. 5D–F). When we explored this region, we observed that CpG island 132 was markedly differentially methylated between CeD patients and controls; the methylated nucleotides of that site coincided with the DNA regulation elements. By using the Epigenome Roadmap tool (http://www.roadmapepigenomics.org/), we identified the presence of several regulation elements as histone modifications H3K27ac and H3K4me3 in the small intestine and H3K4me1, H3K4me3, and H3K9ac in duodenum mucosa and a DNAse hypersensitive tract (Fig. 6).

c-REL and TNFAIP3 in the *lamina propria* showed higher methylation profiles in CeD patients than in controls (Fig. 5G,H–L). C-REL, which contributed to the differential gene expression profiles between CeD patients and controls, showed an inverse methylation profile (Fig. 5I–L,M).

## Discussion

The increased incidence of CeD suggests a new epidemic of the current era. A strong genetic component has been confirmed. However, genes cannot explain a sudden increase in the incidence of a disease.

Several GWASs identified 57genes as associated with CeD, here referred to as “candidate genes”, each giving a small contribution to the CeD genetic risk. Together, these genes account for no more than 15% of the heritability of the disease^7^.Hence, half of the heritability remains to be explained.

None of the associated polymorphisms are in the coding region of the related gene, but at least half of these polymorphisms were related to the regulation of gene expression. We hypothesize that the study of epigenetic mechanisms could provide answers for some of the key questions in this field^15^.

In our previous studies, we explored the expression of candidate genes in the cells and tissues of several cohorts of CeD patients in the diagnostic phase of the disease or after treatment, in those of potential CeD patients and in those of a cohort of at-risk infants from CeD families^13,14^. These studies suggest an interesting scenario of interrelated expression of candidate genes and reinforce the actual selection of a small set of genes putatively implicated in the gluten-induced immune response in several cell compartments. Among the studied “candidate genes”, several genes were found to be differentially expressed in the peripheral monocytes between CeD patients and controls, and we developed multivariate models to discriminate between the two cohorts. We identified a small set of genes that enabled the correct classification (more than 90% correct classification) of the expression of “acute” CeD patients and controls without the need for clinical or serological data. Recently, we studied gene expression in peripheral blood cells in the first year of life and at least one year before the appearance of serum antibodies or clinical complaints. We found that the presence of 5 candidate gene polymorphisms together with the HLA “load” significantly increases the risk of developing the disease. Our findings enabled90% correct prediction of the outcome long before the appearance of any clinical or serological markers^14^. Tissue-related gene expression shows a cellular phenotype of an abnormal response to gluten, and its study may substantially contribute to our understanding of the specific cellular and immune responses to the offending agent in CeD^15^.

Thus, in this study, we explored gene expression in separate cellular compartments comprising a variety of cell types. A “single cell” approach might overcome some of the limitations of our work but will also add complexity to the interpretation of the results.

This is the first work to explore gene expression specifically in the purified epithelial tissue of the small intestine and the “non-epithelial” tissue of the mucosa^16^. Most previous reports present results obtained from isolated cells obtained from whole biopsies via tissue homogenization^17^ or from the isolation of epithelial cells by calcium chelants (EDTA)^18^. Unfortunately, these methods do not prevent cross contamination of different cell types from the epithelium and *lamina propria*.

Our microbead-based separation was efficient, with 98% purity of the epithelial compartment, as verified by RT-PCR. The separation of epithelial cells at high purity from the non-epithelial layer provides novel information about the function (expression) of candidate genes and insight into how the epithelial cells of CeD patients respond to gluten peptides via comparison with the same cells in other subjects that do not recognize gluten peptides.

Our results suggest that genes that are directly (*NFKB*, *IL12A*, *IL21*, and *C-REL)* or indirectly (*SH2B3* and *RGS1*) involved in inflammation or damage processes are significantly up-regulated in CeD patients, in at least one cellular compartment. In contrast, the *PTPRK* gene, which is involved in the maintenance of cell junctions and the inhibition of cell proliferation, was down-regulated in CeD patients relative to controls.

A previous analysis of the correlations among candidate genes by Bilbao &colleagues^16^ showed that the pathway of normal correlation among this set of genes is grossly disrupted in CeD. The genes involved in inflammation do not function synergistically in CeD patients as they do in control subjects. It also appears that when we compared the correlations present in control cells with that observed in the cells of CeD subjects, new patterns appear in CeD: SH2B3 is co-regulated with other genes of inflammation (*TAGAP* ρ = 0.7, IL12 ρ = 0.65, cREL ρ = 0.76, *ARHGAP31* = 0.47) only in CeD. We confirmed the presence of an altered correlation among tight junction genes, as observed by Jauregi-Miguel *et al*., in the mucosa of CeD patients on gluten-containing diets, which was restored after dietary treatment^19^. A good correlation among genes of the NFkB pathway was observed in the controls, whereas a significant disruption of these genes was observed in the CeD patients^16^. In contrast, co-methylation was stronger in CeD patients.

The present study is limited to the estimation of mRNA; hence, our understanding is incomplete since information on protein synthesis and regulation after the production of the messenger remains lacking.

The encouraging results of the differential gene expression analysis prompted us to explore the mechanisms of DNA methylation in the same set of candidate genes. The methylation data suggest modifications of the reading of the individual genome, which are unlikely to occur during the short-term development of the flat mucosa. Such modifications are generally considered to occur in the very early phase, including before birth.

DNA methylation is one of several mechanisms of epigenetic regulation of gene expression. We aimed to explore the regulation of interleukin IL21 and the SH2B3 gene, which play an unique roles in the pathogenesis of CeD. The multivariate discriminant analysis of the epithelium confirmed the pivotal roles of IL12-IL21 (in both cellular compartments) and the SH2B3 gene (in the epithelium). SH2B3 expression is higher in CeD patients than in CTRs in both the small intestine and peripheral blood cells before the appearance of the disease. This gene exerts multiple functions and establishes connections between immunity and inflammation^20^. The SH2B3 gene maps to chromosome12 at 12q24 and encodes a member of the Src homology 2-Binding (SH2-B) protein family, which is described as a negative regulator of T cell receptors, and it is implicated in T cell signalling^21–23^. SH2B3 is also a key regulator of haematopoietic cell lines, being a negative regulator of B cell lymphopoies is in the early phase of development and is expressed in hematopoietic stem cells (HSC) stem and hematopoietic progenitor cells (HPC) progenitor cell lines, the functions of which increase significantly in the absence of SH2B3^22,24^.

SH2B3 is also involved in the three signalling pathways induced by erythropoietin (EPO)and thrombopoietin (TPO), which down-regulate JAK2 and stimulate HSC sand the production of megakaryocytes and erythrocytes. These data support the hypothesis that SH2B3 is a major negative regulator of HSC expansion and the production of blood cells through the modulation of growth factors and cytokines^25–27^. Outside the haematopoietic domain, SH2B3 is expressed in endothelial cells, phosphorylated by TNFa, and rapidly up-regulated either at the mRNA or protein level^28^. The ability of TNF to regulate SH2B3 has also been shown in human umbilical vein endothelial cells^29^.

Recently, it has been suggested that a lack of SH2B3 decreases the precursors of vascular cell adhesion molecule 1 (VCAM-1) on the membrane, suggesting a unique role of this gene in cell motility and adhesion^30,31^.

Despite knowledge of its multiple contributions, the complete pattern of SH2B3 regulation is not yet clear. This study, for the first time, shows that decreased methylation of a gene may modulate over-expression in the epithelium of CeD patients, suggesting an epigenetic regulation of the gene. Bioinformatics analysis showed that the differential methylation is centred in a genomic area of a DNA regulatory element and involves 4key histone modifications and a DNAse hypersensitive tract.

New types of experimental work, such as *in vitro* affinity tests and protein studies, are needed. Regardless, we suggest that the confirmed role of SH2B3 gene is well adapted to the impaired immune regulation observed after the gluten “offence” to CeD mucosa.

SH2B3 over-expression modifies the innate immune response, and the parallel induction of the gene by pro-inflammatory cytokines suggests the development of an inflammation “loop” induced by gluten peptides either at innate or induced levels.

In conclusion, we revealed the differential expression of candidate genes between CeD patients and controls in specific cell compartments of the intestinal mucosa. In addition, we identified a specific “gene expression phenotype” of CeD patients and showed that the abnormal response to dietary antigens might not be essentially related to abnormalities of gene structure but to the fine regulation of the pathways that respond to dietary antigens.

CeD patients appear to be “healthy and normal” people whose response to an abnormal dietary peptide is “physiologically” excessive and leads to inflammation and, eventually, severe cell destruction and clinical disease.

## Methods

### Patient enrolment

Expression studies of candidate genes in cells isolated from intestinal mucosa were performed on biopsy samples of duodenal mucosa from 19 patients (11 F and 8 M, median age 8 years, range 2–16 years) with CeD at the time of diagnosis and 21 (9 F and 12 M, median age 10 years, range 2–16 years) non-CeD CTRs (Supplementary Table 3). Written informed consent was obtained from the parents of the enrolled children, and the study was approved by The Independent Committee for Bioethics of I.R.C.C.S. Burlo Garofolo (Approval Number: CE/V-131). The patients were enrolled in the Department of Gastroenterology, Digestive Endoscopy and Clinical Nutrition of the Burlo Garofolo Hospital of Trieste, and the relevant biopsy samples of duodenal mucosa were collected by esophagogastroduodenoscopy (EGD).

### Purification of intestinal epithelial cells

The biopsy samples were processed immediately after collection, and purification of the intestinal epithelium was performed using enzyme digestion followed by magnetic bead sorting as described previously^32^.

### Extraction of nucleic acids and RNA reverse transcription

Total RNA was extracted from intestinal cells by using the All Prep DNA/RNA Kit Mini Kit (QIAGEN), which enables the simultaneous extraction of DNA and RNA from the same biological sample through a single procedure. The protocol provided by the manufacturer was followed. The kit uses column separation, and two nucleic acids are obtained by means of two different elutions. The quantities of RNA and DNA were measured using a Nanodrop® spectrophotometer, and RNA quality was analysed by agarose gel electrophoresis in Tris/Borate/EDTA buffer (TBE). RNA samples that failed quality control were excluded from further analysis.

The RNA (starting from 1 μg) was transcribed into cDNA by using the High-Capacity Reverse Transcription Kit (Applied Biosystems®).

### Real-time PCR

Real-time PCR was performed by TaqMan methodology (7900T) using TaqMan Gene Expression (Life Technologies) probes. The relative expression of each gene was obtained by using the ΔΔct method and normalized to the expression of an endogenous gene (GUSb) as described elsewhere^33^.

The assays on demand, related to the candidate genes selected in our study, were provided by Applied Biosystems and are listed in Table 1. The candidates were divided into 4 categories based on their biological functions: (1)genes directly involved in inflammation/cell damage, (2) “classical” candidate genes strongly associated with CeD, (3)genes involved in the homing of lymphocytes and the regulation of inflammatory processes and cell damage, and 4)genes involved in cell adhesion and intestinal barrier integrity.

### Methylation analysis

Methylation analysis of intestinal cells was performed for all enrolled subjects. DNA was extracted (as previously described) from the epithelial cells and the *lamina propria* isolated from the biopsy samples.

#### Conversion with bisulfite

The methods developed to detect and quantify DNA methylation use sodium bisulfite, through which unmethylated cytosines are deaminated and sulfonated for conversion into uracil, whereas the 5′-methyl-cytosines remain unchanged. The treatment therefore allows non-methylated cytosines to be discriminated from methylated ones, which are revealed by subsequent analyses. For the sodium bisulfite treatment, the Bisulfite Conversion kit from Active Motif® was used. The obtained samples were subsequently analysed by methylation-specific PCR.

A calibration curve was used to quantify the methylation status of each sample. The curve was constructed by using different concentrations of genomic DNA from HeLa cells, commercially available as both unmethylated and methylated DNA CpG. The following curve concentration levels were used: 0.12, 0.5, 25, 37.5, 50, 62.5, 75, 87.5 and 100%.

#### Methylation-specific PCR (MSP)

Methylation-Specific PCR (MSP) is a widely used technique for studying the methylation of CpG islands. The differences observed after Na-bisulfite treatment between methylated and non-methylated cytosines are at the basis of the functioning of MSP. The primers for MSP were designed by the MethPrimer program (http://www.urogene.org/methprimer/) based on CpG islands in the DNA sequence identified by Genome Browser (Supplementary Table 4).

We designed two sets of primers: one set to recognize sodium bisulfite-modified unmethylated DNA and a second set to identify methylated DNA. Using the Primer3 program, a third set of primers was developed to screen for unmodified DNA and assess the efficiency of bisulfite treatment. Na-bisulfite-treated DNA samples were amplified by three different probes provided with the *KAPA2G Fast HotStartReadyMix PCR Kit* (KAPABIOSYSTEMS®).The amplified samples were analysed by electrophoresis on a 2% agarose gel.

#### DNAclean up

The NucleoMag®96 PCR clean-up kit from MACHEREY-NAGEL® was used to purify the samples.

#### Sequencing

The processed samples were sent for next-generation high-throughput sequencing on an Illumina Nextera platform.

### Statistics

Due to the small sample size, the gene expression levels were compared by Mann-Whitney rank test. Percentages were analysed by Chi-square test, with 1st degree error at 0.05.

To estimate the contribution of the expression of each gene in either the epithelium or the *lamina propria* to the differentiation of cases and controls, we adopted a stepwise discriminant analysis as in previous study^14^. The model was used to estimate the capacity of each gene to discriminate between cases and controls as indicated by Wilks’ Lambda, which ranged from 1 = complete overlap between groups to 0 = complete separation between groups. The variance ratio F was used to evaluate the significance of the contribution of each gene, taking into account the effects of all other genes.

By multiplying the standardized value of gene expression by the respective canonical discriminant coefficient, it was possible to obtain, for each individual, a probability of membership among the cases or the controls.

The percentage of correct classification provided an estimate of the reliability of the model in separating the two groups. Statistical analysis was performed with SPSS 21.0 (SPSS Inc., Chicago, IL).

### Bioinformatics analysis

We analysed the genome sequences containing CpG islands, which were selected in the methylation analysis, using Epigenome Roadmap software^34^.This tool can analyse several key histone modifications, chromatin accessibility, DNA methylation and mRNA expression in a specific tissue or cell population; for this study, we selected small intestine and duodenum mucosa tissues. The Genomics and Proteomics facility of the University of the Basque Country sequenced the amplicons of the selected genes using pair-ended reads and an Illumina MiSeq platform. We employed different approaches to select the most optimal sequences for analysis: we used two mapping programs (BWA and Bowtie).In both cases, the reference sequence was the bisulfite-treated sequence. Bowtie achieved better mapping, and there were no differences between the two references, so we decided to use Bowtie and the “gene as chromosome” reference sequence to map all of the samples. However, when we analysed the percentage of Cs in CpG and no-CpG positions, we decided to take into account a CpG only if its surrounding no-CpGs had a C proportion <0.10. Then, using the CpGs that fulfilled this criterion, we performed Mann-Whitney U-tests between Celiac and No-Celiac values in CD326 positives and negatives.

### Ethics approval and consent to participate

All procedures performed in studies involving human participants were performed in accordance with the ethical standards of the institutional and/or national research committee and with the 1964 Helsinki declaration and its later amendments or comparable ethical standards. Written informed consent was obtained from the parents of the enrolled children, and the study was approved by an independent ethical committee (CE/V-131).

## Supplementary information

COMBINED ANALYSIS OF METHYLATION AND GENE EXPRESSION PROFILES IN SEPARATE COMPARTMENTS OF SMALL BOWEL MUCOSA IDENTIFIED CELIAC DISEASE PATIENTS’ SIGNATURES.

## Data Availability

The datasets used and/or analysed during the current study are available from the corresponding author on reasonable request.
