# Supplementary material for: Combined Analysis of Methylation and Gene Expression Profiles in Separate Compartments of Small Bowel Mucosa Identified Celiac Disease Patients’ Signatures
Source: Sci Rep. 2019 Jul 10;9:10020. doi: 10.1038/s41598-019-46468-2 (PMC6620355; doi:10.1038/s41598-019-46468-2)
Supplement: Supplementary file 1 — COMBINED ANALYSIS OF METHYLATION AND GENE EXPRESSION PROFILES IN SEPARATE COMPARTMENTS OF SMALL BOWEL MUCOSA IDENTIFIED CELIAC DISEASE PATIENTS’ SIGNATURES. [file 41598_2019_46468_MOESM1_ESM.pdf]

SUPPLEMENTARY INFORMATIONS:

COMBINED ANALYSIS OF METHYLATION AND GENE EXPRESSION PROFILES IN SEPARATE COMPARTMENTS OF SMALL BOWEL MUCOSA IDENTIFIED CELIAC DISEASE PATIENTS' SIGNATURES.

Cielo D[12*] & Galatola M[12*], Fernandez-Jimenez N[3], De Leo L[4], Garcia-Etxebarria K[3], Loganes C[4], Tommasini A[4], Not T[3,4], Auricchio R[12], Greco L[12], and Bilbao JR[3].

*These authors contributed equally to this work.

[1] Department of Translational Medical Sciences, University of Naples "Federico II"

[2]European Laboratory for the Investigation of Food Induced Diseases (ELFID), University of Naples "Federico II"

[3]Department of Genetics, Physical Anthropology and Animal Physiology, University of the Basque Country (UPV-EHU), BioCruces Health Research Institute, Leioa, Spain

[4]Institute for Maternal and Child Health, IRCCS "Burlo Garofolo", Trieste, Italy

STEP 0		STEP 1		STEP 2		STEP 3		STEP 4		STEP 5	
**GENE**	**F**	**GENE**	**F**	**GENE**	**F**	**GENE**	**F**	**GENE**	**F**	**GENE**	**F**
SH2B3_EPI	5,95	SH2B3_EPI	2,39	SH2B3_EPI	3,455	SH2B3_EPI	2,74	SH2B3_EPI	3,32	SH2B3_EPI	
TAGAP_EPI	1,08	TAGAP_EPI	0,20	TAGAP_EPI	,145	TAGAP_EPI	0,00	TAGAP_EPI	0,39	TAGAP_EPI	0,24
TNFSF14_EPI	0,02	TNFSF14_EPI	5,28	TNFSF14_EPI		TNFSF14_EPI		TNFSF14_EPI		TNFSF14_EPI	
KIAA_EPI	0,30	KIAA_EPI	0,19	KIAA_EPI	,143	KIAA_EPI	0,05	KIAA_EPI	0,35	KIAA_EPI	0,08
TNFAIP3_EPI	3,79	TNFAIP3_EPI	1,14	TNFAIP3_EPI	,786	TNFAIP3_EPI	0,37	TNFAIP3_EPI	1,95	TNFAIP3_EPI	0,82
RGS1_EPI	5,01	RGS1_EPI	1,20	RGS1_EPI	,649	RGS1_EPI	0,87	RGS1_EPI	1,78	RGS1_EPI	0,98
cREL_EPI	0,79	cREL_EPI	1,08	cREL_EPI	,781	cREL_EPI	0,34	cREL_EPI	1,62	cREL_EPI	0,04
LPP_EPI	0,99	LPP_EPI	0,55	LPP_EPI	,189	LPP_EPI	0,00	LPP_EPI	0,18	LPP_EPI	0,47
TNFRSF14_EPI	2,11	TNFRSF14_EPI	2,36	TNFRSF14_EPI	3,040	TNFRSF14_EPI	2,97	TNFRSF14_EPI		TNFRSF14_EPI	
PTPRK_EPI	0,24	PTPRK_EPI	0,37	PTPRK_EPI	,011	PTPRK_EPI	0,60	PTPRK_EPI	0,01	PTPRK_EPI	0,06
TJP1_EPI	2,13	TJP1_EPI	0,83	TJP1_EPI	,461	TJP1_EPI	0,55	TJP1_EPI	0,97	TJP1_EPI	0,44
ARGHAP_EPI	0,06	ARGHAP_EPI	0,01	ARGHAP_EPI	,200	ARGHAP_EPI	0,07	ARGHAP_EPI	0,80	ARGHAP_EPI	0,21
C1ORF10_EPI	1,14	C1ORF10_EPI	0,00	C1ORF10_EPI	,054	C1ORF10_EPI	0,00	C1ORF10_EPI	0,28	C1ORF10_EPI	0,04
NFKB1_EPI	1,89	NFKB1_EPI	4,17	NFKB1_EPI	4,726	NFKB1_EPI		NFKB1_EPI		NFKB1_EPI	
IL12_EPI	8,22	IL12_EPI	2,44	IL12_EPI	3,376	IL12_EPI	2,89	IL12_EPI	2,43	IL12_EPI	0,64
IL21_EPI	11,04	IL21_EPI		IL21_EPI		IL21_EPI		IL21_EPI		IL21_EPI	

Table S1: variable selection through the stepwise procedure in the Epithelium. The first best gene selected at that step is marked in red, and the second best gene selected at a that step is marked in green.

STEP 0		STEP 1		STEP 2		STEP 3		STEP 4		STEP 5		STEP 6	
**GENE**	**F**	**GENE**	**F**	**GENE**	**F**	**GENE**	**F**	**GENE**	**F**	**GENE**	**F**	**GENE**	**F**
SH2B3_LP	0,87	SH2B3_LP	0,49	SH2B3_LP	,736	SH2B3_LP	0,00	SH2B3_LP	0,13	SH2B3_LP	1,02	SH2B3_LP	0,25
TAGAP_LP	1,74	TAGAP_LP	1,05	TAGAP_LP	,862	TAGAP_LP	0,84	TAGAP_LP	1,62	TAGAP_LP	1,54	TAGAP_LP	0,06
TNFSF14_LP	4,92	TNFSF14_LP	1,78	TNFSF14_LP	3,391	TNFSF14_LP		TNFSF14_LP		TNFSF14_LP		TNFSF14_LP	
KIAA_LP	1,20	KIAA_LP	0,79	KIAA_LP	,185	KIAA_LP	0,06	KIAA_LP	0,74	KIAA_LP	1,91	KIAA_LP	
TNFAIP3_LP	0,59	TNFAIP3_LP	0,02	TNFAIP3_LP	,018	TNFAIP3_LP	0,00	TNFAIP3_LP	0,04	TNFAIP3_LP	0,01	TNFAIP3_LP	0,24
RGS1_LP	4,61	RGS1_LP	0,36	RGS1_LP	3,199	RGS1_LP	4,36	RGS1_LP	2,62	RGS1_LP	1,25	RGS1_LP	0,25
cREL_LP	0,11	cREL_LP	0,12	cREL_LP	,046	cREL_LP	0,02	cREL_LP	0,19	cREL_LP	0,63	cREL_LP	0,05
LPP_LP	0,95	LPP_LP	0,15	LPP_LP	,026	LPP_LP	0,02	LPP_LP	0,01	LPP_LP	0,04	LPP_LP	0,50
TNFRSF14_LP	2,04	TNFRSF14_LP	0,97	TNFRSF14_LP	1,911	TNFRSF14_LP	0,09	TNFRSF14_LP	0,02	TNFRSF14_LP	0,02	TNFRSF14_LP	0,05
PTPRK_LP	6,36	PTPRK_LP	3,59	PTPRK_LP	3,191	PTPRK_LP	5,51	PTPRK_LP	2,66	PTPRK_LP		PTPRK_LP	
TJP1_LP	1,24	TJP1_LP	0,36	TJP1_LP	,029	TJP1_LP	0,02	TJP1_LP	0,01	TJP1_LP	0,07	TJP1_LP	0,22
ARGHAP_LP	0,98	ARGHAP_LP	0,01	ARGHAP_LP	,209	ARGHAP_LP	0,01	ARGHAP_LP	0,15	ARGHAP_LP	0,19	ARGHAP_LP	0,10
NFKB1_LP	5,43	NFKB1_LP	11,45	NFKB1_LP		NFKB1_LP		NFKB1_LP		NFKB1_LP		NFKB1_LP	
IL12_LP	23,10	IL12_LP		IL12_LP		IL12_LP		IL12_LP		IL12_LP		IL12_LP	
C1ORF10_LP	0,02	C1ORF10_LP	0,00	C1ORF10_LP	,342	C1ORF10_LP	1,13	C1ORF10_LP	0,75	C1ORF10_LP	0,03	C1ORF10_LP	0,19
IL21_LP	7,15	IL21_LP	5,37	IL21_LP	1,936	IL21_LP	8,49	IL21_LP		IL21_LP		IL21_LP	

Table S2: variable selection through the stepwise procedure in the Lamina Propria. The first best gene selected at that step is marked in red, and the second best gene selected at a that step is marked in green.

ID	Age	Sex	Diagnosis	anti-tTG U/ml*	EMA**	Histology†	Symptoms
**CD01**	2	F	Celiacdisease	9	+	M3	Abdominal pain
**CD04**	9	M	Celiacdisease	25	+	M2	Abdominal pain
**CD05**	8	F	Celiacdisease	222	+	M3	Arthralgia
**CD07**	8	F	Celiacdisease	142	+	M3	Diarrhea
**CD08**	3	M	Celiacdisease	74	+	M3	Failure to thrive
**CD15**	7	F	Celiacdisease	128	+	M3	Diarrhea
**CD18**	8	F	Celiacdisease	201	+	M3	Failure to thrive
**CD19**	11	M	Celiacdisease	5	+	M3	Abdominal pain
**CD20**	11	F	Celiacdisease	50	+	M3	Adominal pain
**CD23**	7	M	Celiacdisease	21	+	M0	Diarrhea
**CD28**	13	M	Celiacdisease	9	+	M0	Anemia
**CD30**	3	F	Celiacdisease	50	+	M3	Aphtous stomatitis
**CD33**	9	M	Celiacdisease	44	+	M3	Failure to thrive
**CD34**	16	F	Celiacdisease	148	+	M3	Diarrhea
**CD37**	5	F	Celiacdisease	208	+	M3	Aphtous stomatitis
**CD41**	9	F	Celiacdisease	171	+	M3	Anemia
**CD42**	5	F	Celiacdisease	134	+	M3	Abdominal pain
**CD43**	5	M	Celiacdisease	191	+	M3	Anemia
**CD50**	10	M	Celiacdisease	4	+	M1	Failure to thrive
**CTR02**	5	F	Celiacdiseasedenied	**0**	**-**	**M0**	Abdominal pain
**CTR10**	11	M	Eosinophilicesophagitis	**N.A**	**N.A**	**N.A**	N.A
**CTR11**	9	M	Gastritis	**N.A**	**N.A**	**N.A**	N.A
**CTR12**	15	F	Refluxesophagitis	**N.A**	**N.A**	**N.A**	N.A
**CTR16**	9	M	Celiacdiseasedenied	**0**	**-**	**M0**	Abdominal pain
**CTR17**	9	M	functionalabdominalpain	**N.A**	**N.A**	**N.A**	N.A
**CTR21**	3	M	Causticingestion	**N.A**	**N.A**	**N.A**	N.A
**CTR24**	15	M	Refluxesophagitis	**N.A**	**N.A**	**N.A**	N.A
**CTR25**	7	F	Celiacdiseasedenied	**2**	**-**	**M0**	Abdominal pain
**CTR27**	15	M	Celiacdiseasedenied	**1**	**-**	**M0**	Abdominal pain
**CTR29**	9	F	Gastritis	**N.A**	**N.A**	**N.A**	N.A
**CTR32**	13	M	Eosinophilicesophagitis	**N.A**	**N.A**	**N.A**	N.A
**CTR36**	2	F	Celiacdiseasedenied	**0**	**-**	**M0**	Constipation
**CTR38**	9	F	Celiacdiseasedenied	**1**	**-**	**M0**	Abdominal pain
**CTR39**	12	M	Refluxesophagitis	**N.A**	**N.A**	**N.A**	N.A
**CTR40**	16	F	Suspected Autoimmune Poliendocrine Syndrome	**0**	**-**	**M0**	N.A
**CTR44**	11	F	Gastritis	**N.A**	**N.A**	**N.A**	N.A
**CTR45**	10	M	Celiacdiseasedenied	**0**	**-**	**M0**	Abdominal pain
**CTR46**	12	M	Eosinophilicesophagitis	**N.A**	**N.A**	**N.A**	N.A
**CTR48**	8	F	Celiacdiseasedenied	**0**	**-**	**M0**	Abdominal pain
**CTR49**	13	M	Celiacdiseasedenied	**0**	**-**	**M0**	Diarrhea

Table S3. Patients enrolled in the study. N.A. Not Available* For the diagnosis of CD Marsh classification has been applied, all biopsed controls have a normal duodenal mucosa with no atrophy (Marsh lesion stage M0-M1).**EMA:Anti-Endomysial Antibody†Reference values of anti-tTG: Negative: < 4.0 U / mL; Doubt: > 4.0 and < 10.0 U / mL; Positive: > 10.0 U / mL.

PositionUCSC (GRCh37/hg19)	Chr	Locus	Length	CpG Island	CpG Island Length	Met-Primers Sequence	Amplicon Length
61108026-61109316	2	cREL	1291 bp	80	757 bp	FP-GGTTTTTTGATTGATTGATTG<br>RP-CCATATAAAATTTTAAAAACAAAAC	216bp
88706112-88706792	16	IL21	681 bp	30	305bp	FP-TTTAGGAGGATAGGGTAGGGTTAGA<br>RP-AACAAAAACCCCAAATATAAAAAAC	247 bp
103421992-103423529	4	NFKB1	1538 bp	100	207 bp	FP-GGAGGTTGATAGTAGTTGAGAGGTATAT<br>RP-CATCCCCCAAATAAAACTAAAATTAA	205 bp
200859977-200860876	1	C1orf106	900bp	53	248 bp	FP-GGTATTGGGATTAAGAGAGAAGTTT<br>RP-TTTACAACATCCCAAATATTAAAAATC	300bp
128840059-128842426	6	PTPRK	2368bp	163	847 bp	FP-TTTTTTTATTTTGTGTTTTTTATTTT<br>RP-ACTCCTCTCTCCTTAACCTCTCCTA	192 bp
159706391-159707311	3	IL12A	921 bp	52	137 bp	FP-TAAAAATGTGGTTTTTTGGGTTAGT<br>RP-ACAAACTTAAATTTCTACCCACTCTTC	209 bp
111842839-111844692	12	SH2B3	1854 bp	132	796 bp	FP-TATTTTTTTTAGAATTGGGGGATAG<br>RP- CCCTCCTACAAAACCTTATCAATAC	260 bp
30114011-30115515	15	TJP1	1505 bp	124	1505 bp	FP-TATGGGGTGAGTTTTATGTATGAGT<br>RP-ATCACCAATTTCAACCTTAACAATC	262 bp
123300308-123301105	4	KIAA1109	798 bp	45	399 bp	FP -TTATTTGGTTTTTTTAGAAGGGTAAG<br>RP -AAATTCACAAAATCAAAACC	287 bp
187871379-187872654	3	LPP	1276 bp	67	448 bp	FP-TTTTTTTATATTTTATATGGATAAATG<br>RP-TACAACCAACCCTTACCTTACAAC	286 bp
137867116-137868004	6	TNFAIP3	889 bp	82	766 bp	FP -TTATTTGGTTTTTTTAGAAGGGTAAG<br>RP -AAATTCACAAAATCAAAACC	300 bp

Table S4. The primer summary table contains RefSeq Human gene to visualize in UCSC genome browser, CpG islands and related length, forward and reverse primer sequence.

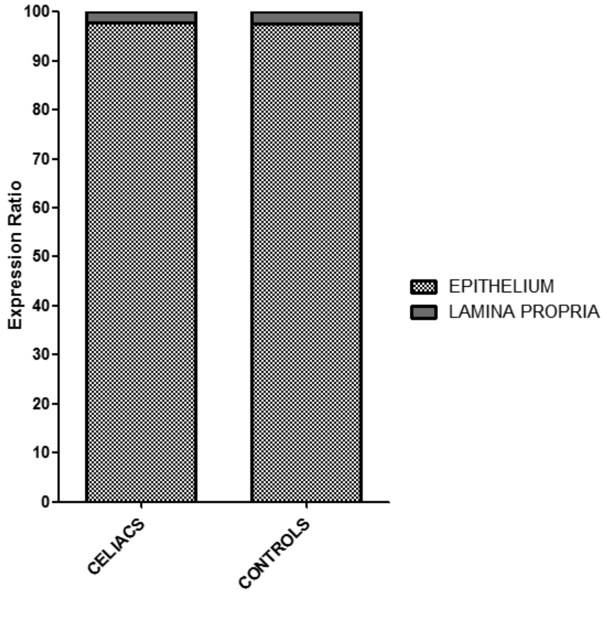

Supplementary Figure 1: Visualization of the enrichment of CD324+ cells.(A) Expression levels analysis of EpCAM, indicate a 97.8% positive selection rate of CD346+ cells in celiac biopsy samples, and a 97.5% enrichment in controls biopsy samples. The expression levels of EpCAM -expressed transcripts are markedly reduced in CD346- samples.

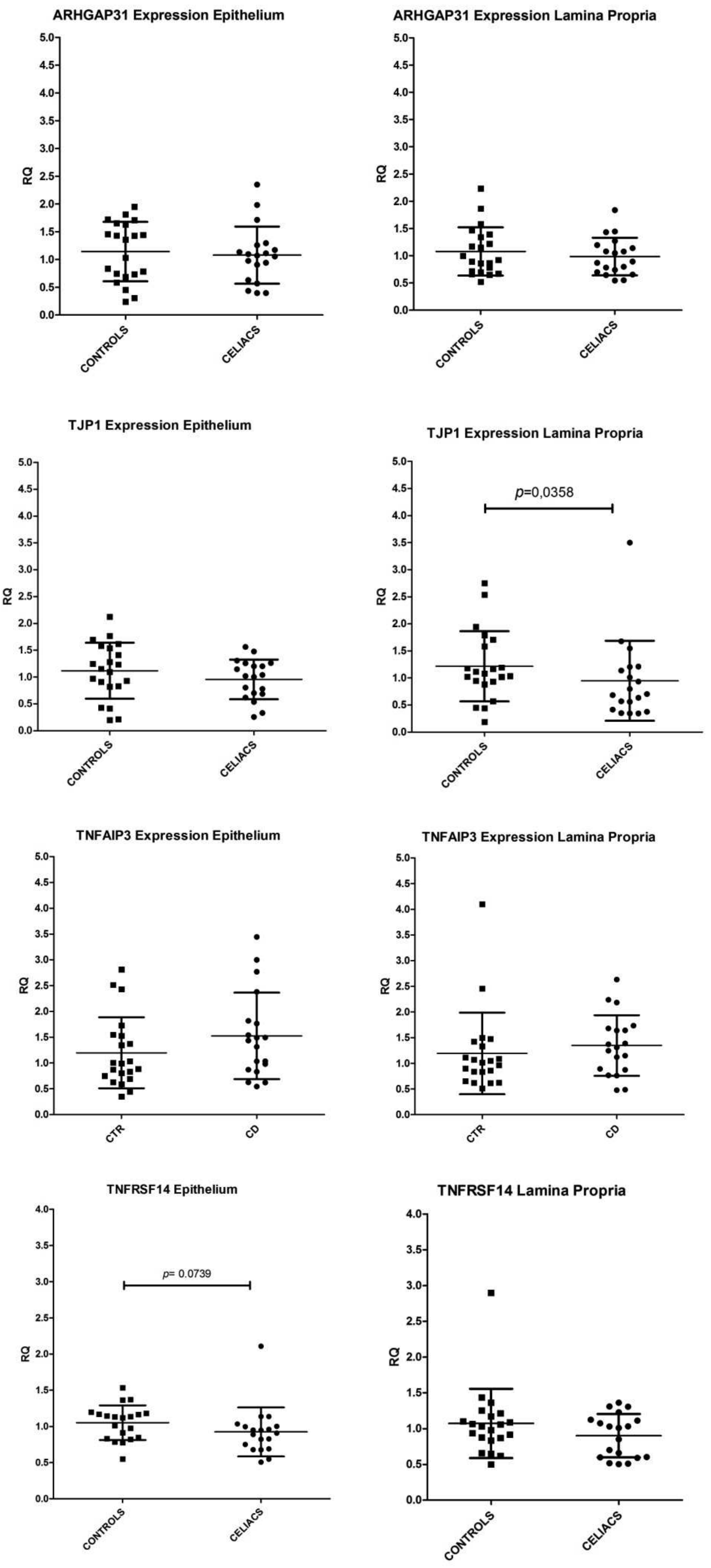
ARHGAP31 Expression Epithelium
RQ
CONTROLS
CELIACS
ARHGAP31 Expression Lamina Propria
RQ
CONTROLS
CELIACS
TJP1 Expression Epithelium
RQ
CONTROLS
CELIACS
TJP1 Expression Lamina Propria
p=0,0358
RQ
CONTROLS
CELIACS
TNFAIP3 Expression Epithelium
RQ
CTR
CD
TNFAIP3 Expression Lamina Propria
RQ
CTR
CD
TNFRSF14 Epithelium
p= 0.0739
RQ
CONTROLS
CELIACS
TNFRSF14 Lamina Propria
RQ
CONTROLS
CELIACS

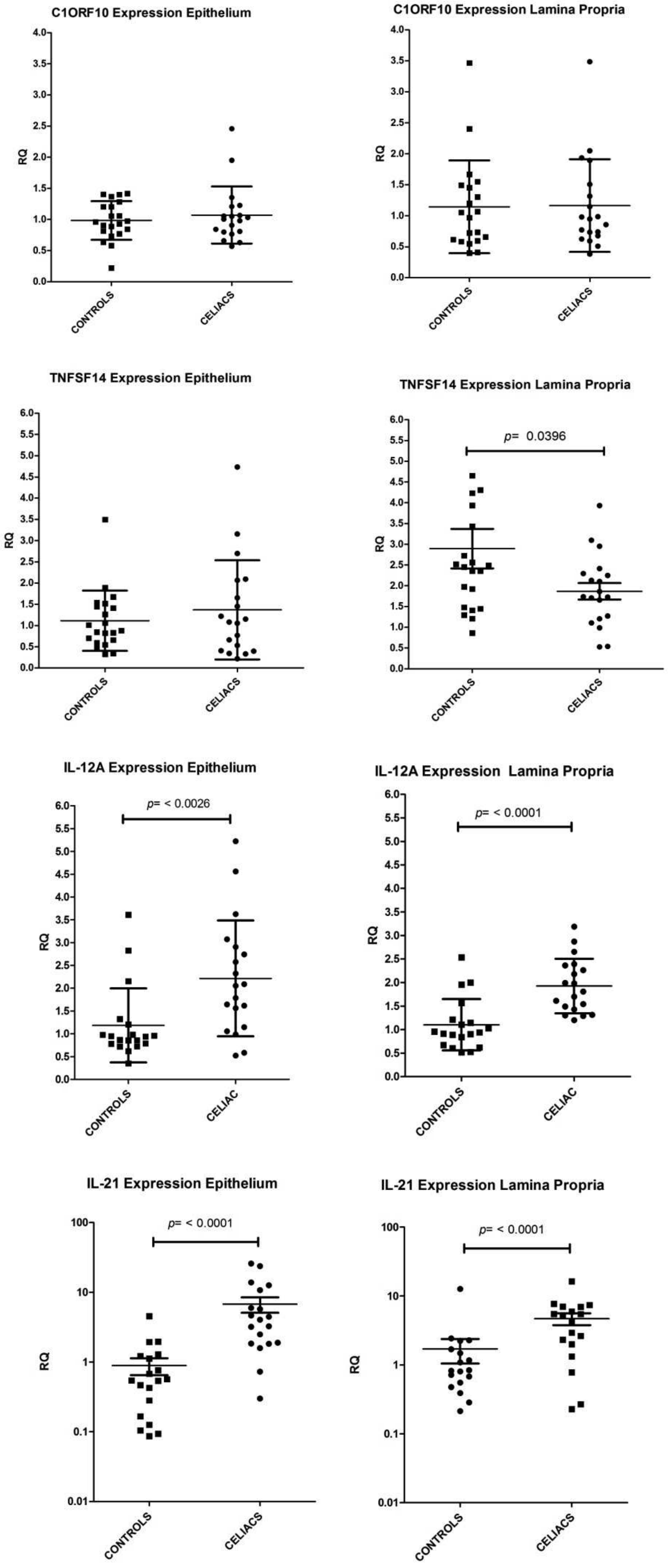
C1ORF10 Expression Epithelium
RQ
CONTROLS
CELIACS
C1ORF10 Expression Lamina Propria
RQ
CONTROLS
CELIACS
TNFSF14 Expression Epithelium
RQ
CONTROLS
CELIACS
TNFSF14 Expression Lamina Propria
p= 0.0396
RQ
CONTROLS
CELIACS
IL-12A Expression Epithelium
p= < 0.0026
RQ
CONTROLS
CELIAC
IL-12A Expression Lamina Propria
p= < 0.0001
RQ
CONTROLS
CELIAC
IL-21 Expression Epithelium
p= < 0.0001
RQ
CONTROLS
CELIACS
IL-21 Expression Lamina Propria
p= < 0.0001
RQ
CONTROLS
CELIACS

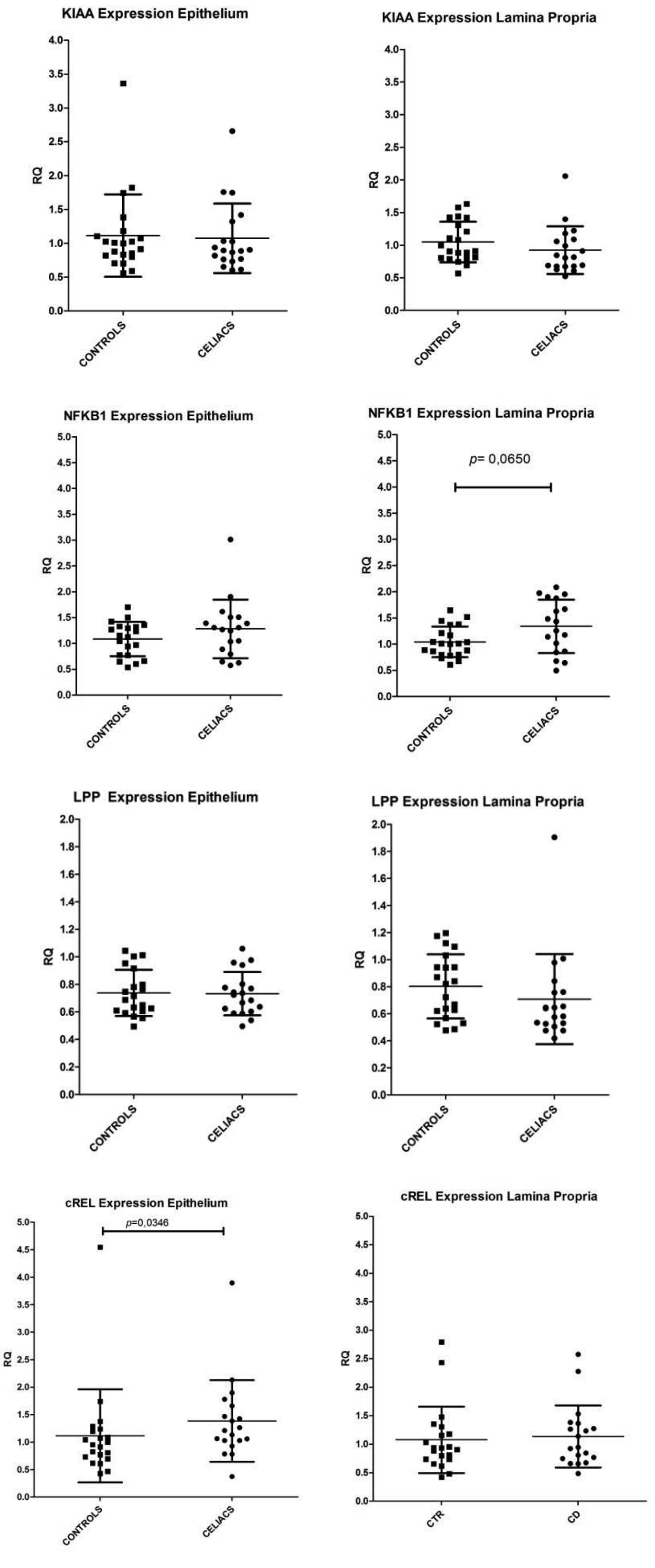

Supplementary Figure 2: Relative expression in Epithelial (left) and Lamina Propria (Right) cells of children CD and control children. The significant difference are indicated in the graphs with the *p* value. RQ: Relative Quantification.

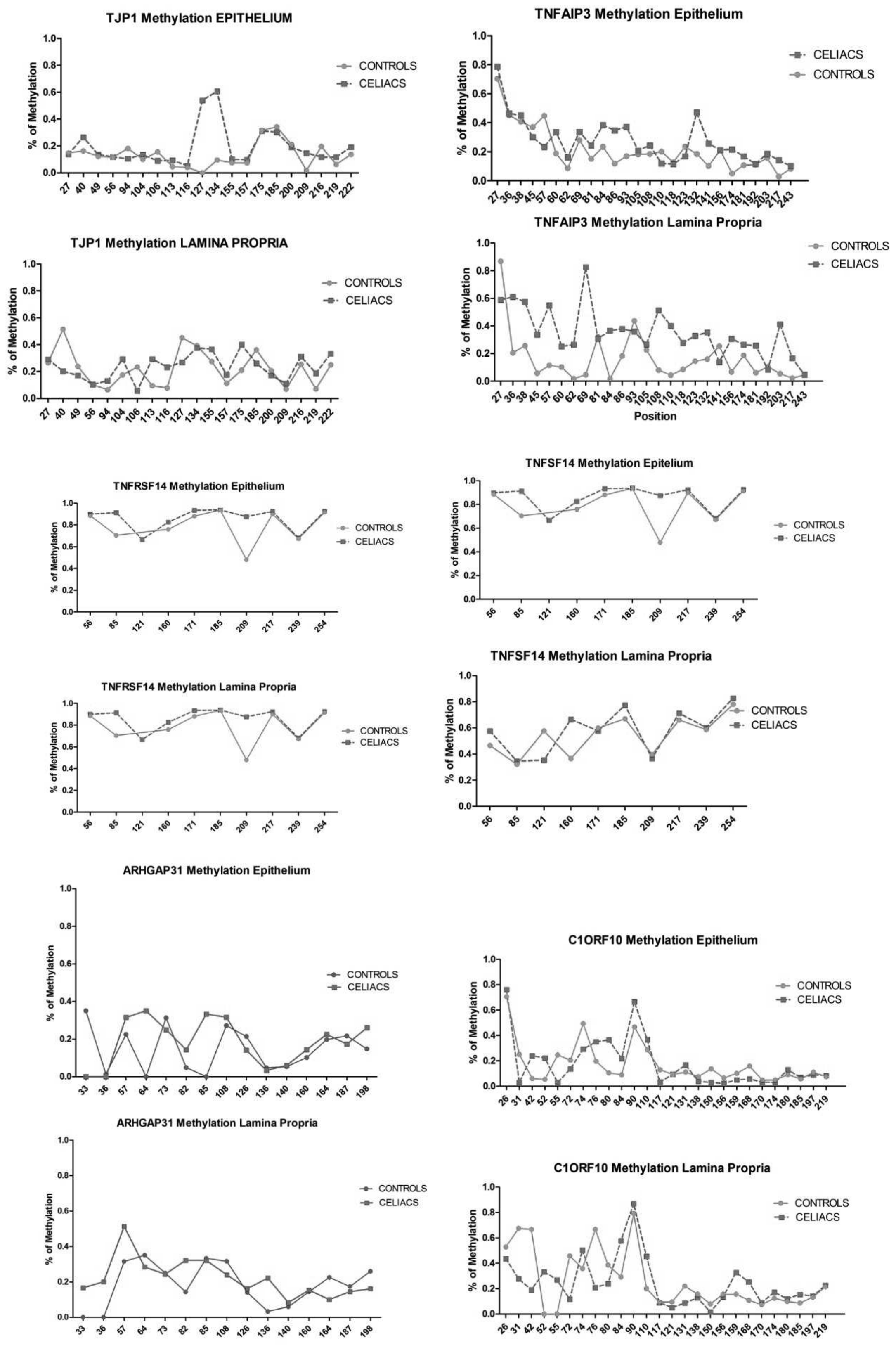
TJP1 Methylation EPITHELIUM
% of Methylation
CONTROLS
CELIACS
TNFAIP3 Methylation Epithelium
% of Methylation
CELIACS
CONTROLS
TJP1 Methylation LAMINA PROPRIA
% of Methylation
CONTROLS
CELIACS
TNFAIP3 Methylation Lamina Propria
% of Methylation
Position
CONTROLS
CELIACS
TNFRSF14 Methylation Epithelium
% of Methylation
CONTROLS
CELIACS
TNFSF14 Methylation Epitelium
% of Methylation
CONTROLS
CELIACS
TNFRSF14 Methylation Lamina Propria
% of Methylation
CONTROLS
CELIACS
TNFSF14 Methylation Lamina Propria
% of Methylation
CONTROLS
CELIACS
ARHGAP31 Methylation Epithelium
% of Methylation
CONTROLS
CELIACS
C1ORF10 Methylation Epithelium
% of Methylation
CONTROLS
CELIACS
ARHGAP31 Methylation Lamina Propria
% of Methylation
CONTROLS
CELIACS
C1ORF10 Methylation Lamina Propria
% of Methylation
CONTROLS
CELIACS

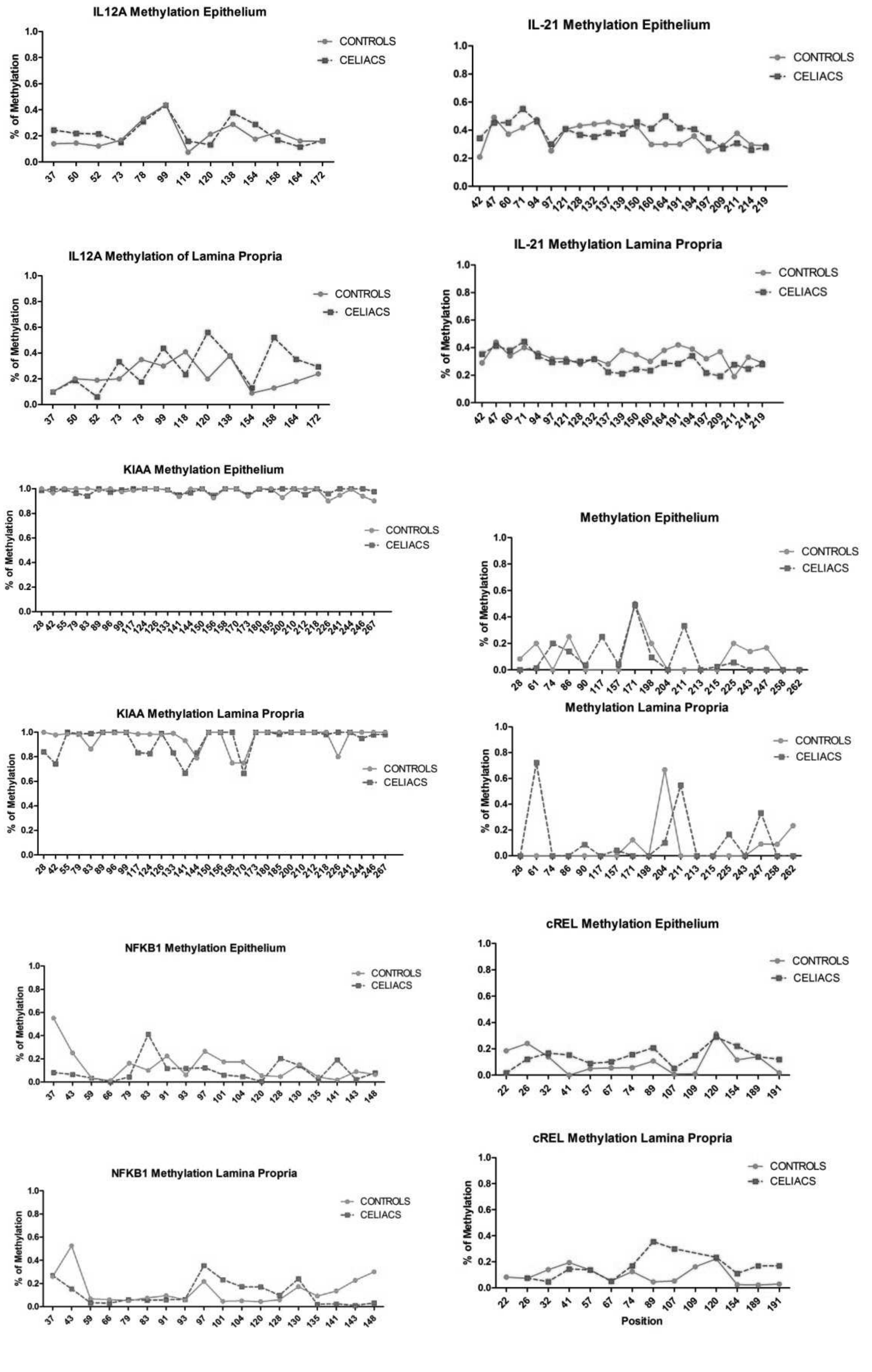

Supplementary Figure 3: Methylation Analysis of candidate genes in epithelial and Lamina propria cells.
